# The Role of Circulating CD16+CD56+ Natural Killer Cells in the Screening, Diagnosis, and Staging of Colorectal Cancer before Initial Treatment

**DOI:** 10.1155/2019/7152183

**Published:** 2019-09-17

**Authors:** Feng Cui, Di Qu, Ruya Sun, Han Tao, Junru Si, Yuqing Xu

**Affiliations:** Department of Oncology, 2nd Affiliated Hospital of Harbin Medical University, China

## Abstract

**Background and Objective:**

A reliable noninvasive prediction tool for the screening, diagnosis, and/or staging of colorectal cancer (CRC) before surgery is critical for the choice of treatment and prognosis.

**Methods:**

Patients admitted for initial treatment of CRC between January 1, 2015, and December 31, 2018, were retrieved and reviewed. Records of CD16+CD56+ natural killer (NK) cells were analyzed according to the stages of CRC.

**Results:**

The number of qualified participants in the healthy, stage I, stage II, stage III, and stage IV CRC patients were 60, 66, 60, 70, and 68, respectively. There was a significant difference in circulating CD16+CD56+ NK cells between the healthy group and the CRC group (*p* < 0.01), as well as between the healthy group and stage III or IV CRC group (*p* < 0.01 and 0.001, respectively). The percentage of circulating CD16+CD56+ NK cells in lymphocytes was negatively correlated with the occurrence of CRC. When comparing the pool of stage I and II CRC cases with the pool of stage III and IV CRC cases using circulating CD16+CD56+ NK cells, the area under the Receiver Operating Characteristic curve was 0.878. Using an optimal cutoff value of 15.6%, the OR was 0.06 (0.03, 0.11), *p* < 0.001, sensitivity was 86.5%, specificity was 72.5%, positive predictive value was 74.2%, and negative predictive value was 85.5%.

**Conclusions:**

Circulating CD16+CD56+ NK cells can be used as a screening and diagnostic/staging tool for CRC.

## 1. Introduction

Colorectal cancer (CRC) has an incidence of about one million per year and causes the death of nearly 700,000 people each year, ranking it the fourth most deadly cancer in the world [1, 2]. The present screening strategy of CRC is faced with low sensitivity and/or specificity in stool-based tests [3], tedious bowel preparation steps before radiographic exams, and high risk of perforation in endoscopic exams [4]. In fact, the best screening and follow-up test with high compliance for CRC should be easily completed and repeated, especially considering the up to 25% unresectable cases at the time of diagnosis and 50% recurrence rate in early-stage cases following surgery [5].

The staging and prognosis of CRC rely mainly on pathology after surgical procedures [6]. A consensus immunoscore on paraffin sections for the classification and prognosis of CRC was a practical example [7]. Although several studies have employed complementary and noninvasive biomarkers in the diagnosis of CRC [8], a reliable prediction tool with high sensitivity as well as specificity for the diagnosis and/or staging of CRC before surgery is still lacking.

The immune system is known to be involved in the development and progression of CRC [9]. Immune infiltration of different immune cells in CRC has been shown to be related to metastasis and prognosis [10]. Furthermore, the circulating immune cells may reflect the local immune response in the tumor microenvironment [11], thereby providing potentially important information regarding disease progression in CRC [12]. Natural killer (NK) cells, as an important subset of the immune cells, whose activity is triggered by an evolving and delicate equilibrium between activating and inhibitory signals received by cell surface receptors, are considered interesting targets for translational and clinical studies [13].

In the present study, we analyzed CD16 and CD56 double positive NK cells in the healthy and different stages of CRC patients before initial treatment, trying to figure out the value of CD16+CD56+ NK cells in the prediction and pretreatment staging of CRC.

## 2. Methods

This was a retrospective cohort study conducted at the 2^nd^ Affiliated Hospital of Harbin Medical University, a tertiary hospital in Northeast China. Institutional Ethics Committee approval was obtained before data collection, and informed consent was obtained from patients on admission.

Clinical records of patients who were admitted for initial treatment of CRC between January 1, 2015, and December 31, 2018, to the Department of Oncology were retrieved and reviewed. Included patients should have pretreatment NK cell data available (the most recent one before the first surgery), as well as histologically confirmed primary CRC. Staging was based on the Tumor Node Metastasis (TNM) terminology [14]. Patients with unclear diagnosis, complicated with other cancers, were admitted after previous treatments for CRC, with other chronic diseases (such as cardiovascular diseases and endocrine diseases), or with viral or bacterial infections were excluded. Age- and BMI-matched healthy participants (no clinical complain who just completed annual physical exam at the time of enrollment) were enrolled in the control group.

Fasting peripheral venous blood samples were collected from all participants before treatment (for the CRC group) or on the day of the annual exam (for healthy controls) in a heparin-coated tube and kept at 2-8°C. 100 *μ*l of freshly collected blood was transferred into a flow-specific tube. 20 *μ*l of a BD Multitest 6-color TBNK reagent (Ref # 644611, BD, USA, including CD3 FITC, CD16 PE+CD56 PE, CD45 PerCP-Cy5.5, CD4 PE-Cy7, CD19 APC, and CD8 APC-Cy7) was added for flow cytometry study according to the manufacture manual, within the panel of which CD16+CD56+ specifically quantified NK cells within the lymphocyte population. The mixture was incubated at room temperature for 15 minutes in the dark and treated with 2.5 ml of red blood cell lysis buffer (Sigma-Aldrich) for 10 minutes at room temperature in the dark. The mixture was washed twice with PBS buffer, resuspended and fixed in 0.5 ml PBS containing 0.9% formaldehyde (Sigma-Aldrich), and analyzed using a FACSCanto II flow cytometer system (BD Biosciences) by FACSDiva™ software version 8.0 (BD Biosciences). At least 20,000 cells were analyzed in P1 gate from each sample.

### 2.1. Statistical Analysis

Discrete data were expressed as the number of cases (percentages) and analyzed using *χ*^2^ test or Fisher's exact test, along with odds ratio (OR) and 95% confidence interval (95% CI), whichever was applicable. Continuous data were shown as the mean ± standard deviation (SD) and were analyzed using the *t*-test. Area under the Receiver Operating Characteristic (ROC) curve was used to show the value of prediction. SPSS 24.0 (IBM Corp., Armonk, NY) was used for statistical analysis. A two-tailed *p* < 0.05 is considered significantly different.

## 3. Results

During the preset study period, 2,714 CRC patients were admitted to our hospital. According to the preset inclusion criteria, 66 of stage I, 60 of stage II, 70 of stage III, and 68 of stage IV patients were included in our study. Another 60 age- and BMI-matched healthy participants were enrolled in the control group. There were no significant differences in age, gender, body weight, height, or BMI between healthy controls and the CRC cases or among different groups (*p* > 0.05, Table 1).

### 3.1. The Predictive Value of Circulating CD16+CD56+ NK Cells in CRC

There was a significant difference in circulating CD16+CD56+ NK cells between the healthy group and CRC cases (*p* < 0.01, Table 1). The percentage of circulating CD16+CD56+ NK cells in lymphocytes was negatively correlated with the occurrence of CRC.

**Figure 1:**
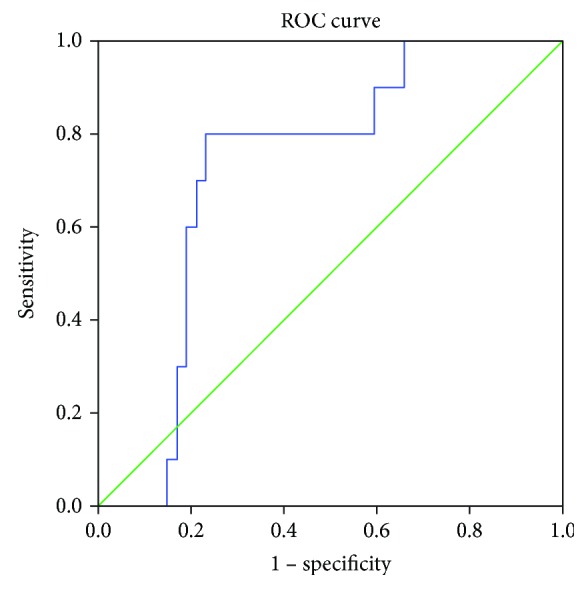
Receiver Operating Characteristic (ROC) curve of NK cells in the prediction of CRC cases (healthy vs. cases).

The Receiver Operating Characteristic (ROC) curve was employed to show the role of CD16+CD56+ NK cells in the prediction of CRC. When comparing healthy controls with CRC cases as a whole (Figure 1), the area under the curve (AUC) was 0.725 (Table 2). Using an optimal cutoff value of 19.7%, the OR was 0.08 (0.04, 0.15), *p* < 0.001, sensitivity was 80.0%, specificity was 76.9%, positive predictive value (PPV) was 44.0%, and negative predictive value (NPV) was 94.4%.

### 3.2. Circulating CD16+CD56+ NK Cells in Different Stages of CRC Cases

**Figure 2:**
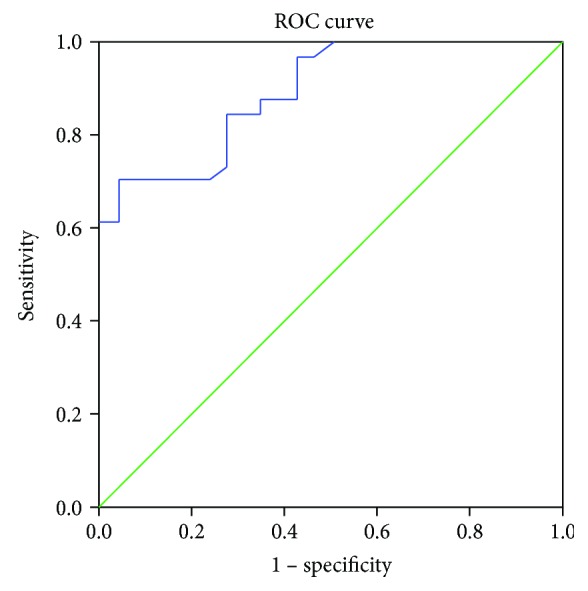
Receiver Operating Characteristic (ROC) curve of NK cells in different stages of CRC cases (healthy+stages 1&2 vs. stages 3&4).

There were no differences in circulating CD16+CD56+ NK cells between the healthy group and stage I or II CRC group (*p* = 0.38 and *p* = 0.28, respectively), but significant differences exist between the healthy group and stage III or IV CRC group (*p* < 0.01, *p* < 0.01, and *p* < 0.001, respectively, Table 1). Since there were no differences in CD16+CD56+ NK cells between the healthy group and stage I or II CRC group, when comparing the pool of healthy controls+stage I+II CRC cases with the pool of stage III+IV CRC cases (Figure 2), the AUC was 0.892 (Table 2). Using an optimal cutoff value of 15.6%, the OR was 0.07 (0.04, 0.12), *p* < 0.001, sensitivity was 84.4%, specificity was 72.5%, PPV was 80.5%, and NPV was 77.5%.

**Figure 3:**
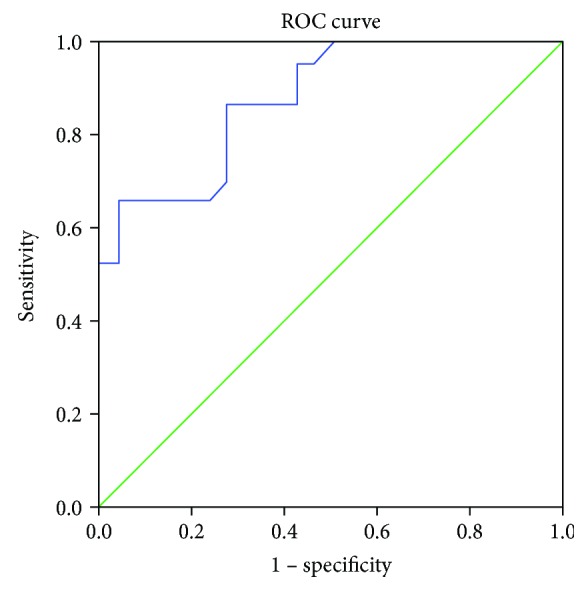
Receiver Operating Characteristic (ROC) curve of NK cells in different stages of CRC cases (stages 1&2 vs. stages 3&4).

When comparing the pool of stage I and II CRC cases with the pool of stage III and IV CRC cases (Figure 3), the AUC was 0.878 (Table 2). Using an optimal cutoff value of 15.6%, the OR was 0.06 (0.03, 0.11), *p* < 0.001, sensitivity was 86.5%, specificity was 72.5%, PPV was 74.2%, and NPV was 85.5%.

## 4. Discussion

Broadly speaking, based on their CD56 expression, NK cells can be subdivided into CD56^bright^ and CD56^dim^. The former cells are associated with immunoregulation and production of proinflammatory cytokines, while the latter cells are cytotoxic [15]. CD16 (Fc*γ*RIII) on NK cells mediates antibody-dependent cell-mediated cytotoxicity [16], and the presence of CD16 thus excludes the involvement of certain NK cells correlated with T or B cells [17].

Recently, two studies used CD3-CD56+ as markers for NK cells. One study reported the presence of subsets of NK cells in CRC patients, with a conclusion of “percentage of CD16+ NKT-like cells was independently associated with shorter disease-free survival in CRC patients” [18]. However, the authors only enrolled a limited number of patients, and the stratification by different stages as well as a split of CD56^bright^ and CD56^dim^ NK cell population further diluted the power of their data. Moreover, some of those patients have received radiological treatment already before collection of NK cells, which might introduce heterogeneity (a mixture of initially treated and posttreatment patients) into the pooled patient population. Another study reported a negative correlation between peripheral NK cells and the TNM staging of CRC, with a significant difference in NK cells between the healthy and stages I and II, and healthy and stage IV, but not between healthy and stage III [19]. This inconsistent trend might be due to the small number of enrolled patients in each group. In our study, we collected more patients, and only patients without previous treatment were enrolled for analysis, which might show the natural body condition under the burden of CRC. Thus, our data, as homogeneous as such, is applicable as a screening test before initial treatment.

Another power of our study resides in the exclusion of viral or bacterial infection cases. There are activating receptors and inhibitory receptors on the surface of NK cells [20]. NK cell-activating receptors, as were exemplified by Ly49H, KIR2DL3, or KIR3DS1, are necessary to clear cytomegalovirus [21], hepatitis C virus [22], or Epstein-Barr virus [23] infections, respectively. On the other hand, NK cell inhibitory receptors, as were exemplified by CD94-NKG2A [24], or KIRs [25], are also involved in the case of viral infection. The balance between activating and inhibitory receptors is maintained by means of receptor-ligand binding [26]. Direct Toll-like receptors (TLRs) can be activated by lipopolysaccharide, a component of gram-negative bacteria. The stimulation of TLRs on NK cells has also been reported to be involved in NK cell activation [27]. Therefore, exclusion of viral or bacterial infection cases will reduce heterogeneity of those cases in the analysis of CRC cases.

There have been reports of the prognosis of CRC based on immunohistochemistry staining to detect gene mutation or polymorphism [28, 29], or activation [30], which is only feasible after surgical procedures. One category of circulating biomarkers for CRC falls within the scope of gene regulation (microRNA or methylation) [31, 32]. Another category resides in serum metabolomics analysis [33]. In this sense, the circulating CD16+CD56+ NK cells have a predictive role in both screening and staging before surgical procedures.

## 5. Conclusion

In summary, we found that the percentage of circulating CD16+CD56+ NK cells was negatively correlated with the occurrence of CRC and the staging of CRC. Using a cutoff value of 19.7% and 15.6%, the percentage of circulating CD16+CD56+ NK cells was able to differentiate between healthy and CRC cases or stage I+II and III+IV cases, respectively.

## Figures and Tables

**Table 1:** Clinical characteristics of enrolled participants.

Cases	Healthy control (*n* = 60)	Stage I (*n* = 66)	Stage II (*n* = 60)	Stage III (*n* = 70)	Stage IV (*n* = 68)	*p* value
Male	34	36	30	39	32	0.40^#^
*p* value^$^	0.50^$$^	0.81	0.446	0.91	0.28	—
Age (years)	54.2 ± 3.5	56.0 ± 11.4	54.5 ± 10.3	56.1 ± 10.0	53.2 ± 15.4	0.49^∗^
*p* value^∗∗^	0.63^$$^	0.25	0.83	0.17	0.59	—
Body weight (kg)	66.8 ± 11.1	70.0 ± 13.1	67.5 ± 7.2	67.3 ± 7.4	67.1 ± 16.1	0.53^∗^
*p* value^∗∗^	0.48^$$^	0.15	0.67	0.77	0.74	—
Height (cm)	168.9 ± 8.2	169.5 ± 8.9	168.0 ± 5.4	167.6 ± 6.9	170.0 ± 9.3	0.40^∗^
*p* value^∗∗^	0.89^$$^	0.72	0.48	0.31	0.45	—
BMI	24.0 ± 3.0	24.3 ± 3.8	24.0 ± 2.7	23.4 ± 3.5	23.5 ± 2.9	0.37^∗^
*p* value^∗∗^	0.56^$$^	0.65	0.94	0.27	0.39	—
Total NK cells (% in lymphocytes)	19.2 ± 5.8	20.7 ± 11.1	21.0 ± 11.2	15.7 ± 8.5	14.7 ± 7.1	<0.001^∗^
*p* value^∗∗^	<0.01^$$^	0.38	0.28	<0.01	<0.001	—

^#^ *χ* ^2^ test among all groups. ^∗^ANOVA test among all groups. ^$^*χ*^2^ test between the healthy control group and the other corresponding groups. ^∗∗^*t*-test between the healthy control group and the other corresponding groups. ^$$^*p* value of healthy vs. CRC cases, *χ*^2^ test or *t*-test.

**Table 2:** The cutoff value of CD16+CD56+ NK cells in prediction of different CRC stages.

Groups	AUC	Threshold (% in lymphocytes)	Case numbers^∗^	OR (95% CI)	*p* value	Sensitivity (%)	Specificity (%)	PPV (%)	NPV (%)
Healthy vs. cancer	0.725	19.7	48/60: 61/264	0.08 (0.04, 0.15)	<0.001	80.0	76.9	44.0	94.4
Healthy+stages 1&2 vs. stages 3&4	0.892	15.6	157/186: 38/138	0.07 (0.04, 0.12)	<0.001	84.4	72.5	80.5	77.5
Stages 1&2 vs. stages 3&4	0.878	15.6	109/126: 38/138	0.06 (0.03, 0.11)	<0.001	86.5	72.5	74.2	85.5

AUC: area under the curve. ^∗^Case number above the threshold/total case number in corresponding groups.

## Data Availability

Original data could be obtained by contacting the corresponding author.
